# Supplementary material for: Chromothripsis during telomere crisis is independent of NHEJ, and consistent with a replicative origin
Source: Genome Res. 2019 May;29(5):737–49. doi: 10.1101/gr.240705.118 (PMC6499312; doi:10.1101/gr.240705.118)
Supplement: Supplemental Material [file supp_gr.240705.118_Supplemental_file_1.zip › contigs/annotated_contigs/DB90/contig.2.DB90_length_419_mean_cov_7.70883054893.docx]

**DB90_length_419_mean_cov_7.70883054893**

```
ACACACACCACATACACCATATATACAC|ACAATACATACATACTACTCAAGACATATCATACCACATACACACCACACACAGCATATA
 >chr13:31416763-31416791 - E=3e-05 p=0e+00
TATACACAATACACACACAATGCATACACACAACACAAGACATATCAT|ACCACACACACAATACATACAATACACCACACATACATAC
                                                 >chr8:54506571-54506854 + E=6e-159
ATATAACATGCACAACACACCACCTACAAACCCACACAACACATACACATGCTACACAAAACATACATGTCCACACACACCACCCACAC

AAATATACTCACCACACACACAAACACAGCAATGCCCCACCCACAGTGGCTGCGATGGTTCCTGCATTTCTCTTTAGTCTCTCGCAGCC

CTGCAGCCGGTCCTGAGCGTTACTCAGCTGGGGGATAACAGACCTGATGCGTCCGCCTCCAGCCC
```
